# Rescuing a “hopeless” tooth with severe internal resorption during orthodontic therapy: a case report

**DOI:** 10.3389/froh.2025.1604976

**Published:** 2025-08-14

**Authors:** Wenjing Liu, Xuezhong Xu, Junqiang Wang, Zhidan Li

**Affiliations:** ^1^ Yan'an Medical College of Yan'an University, Yan'an, China; ^2^ Yan'an University Affiliated Hospital, Yan'an, China; ^3^ Yiwu Zhongya Dental Clinic, Yi’wu, China; ^4^ Key Laboratory of Shaanxi Province for Craniofacial Precision Medicine Research, College of Stomatology, Xi’an Jiaotong University, Xi’an, China; ^5^ Clinical Research Center of Shaanxi Province for Dental and Maxillofacial Disease, College of Stomatology, Xi’an Jiaotong University, Xi’an, China; ^6^ Department of Cariology & Endodontics, College of Stomatology, Xi’an Jiaotong University, Xi’an, China

**Keywords:** root resorption, OIIRR, MTA, internal root resorption, root canal treatment

## Abstract

Root resorption is one of the leading complications that follows orthodontic treatment. It's an inflammatory process involving ischemic necrosis. Therefore, it is called orthodontically induced inflammatory root resorption (OIIRR). The purpose of this report is to present a case study of a patient in her 20s who experienced internal root resorption on two maxillary central incisors as a result of orthodontic treatment. Mineral trioxide aggregates (MTAs) were applied to the root resorption lesion to promote remineralization. A clinical and radiographic examination revealed that no radiolucency related to the resorptive lesion was found without any pathological symptoms after a 3-year follow-up.

## Introduction

Resorption is the progressive physiologic or pathologic loss of dentin, cementum, and/or bone. Root resorption most commonly occurs because of inflammation caused by mechanical, chemical, or thermal injury, such as periodontal treatment, orthodontic tooth movement, and tooth whitening (1). Generally, root resorption can be categorized as internal and external root resorption. Internal root resorption (IRR) is characterized by the loss of the tooth root wall, which is a clinical complication of orthodontic treatment and can lead to tooth extraction. Histologic studies reported a greater than 90% occurrence of orthodontically induced inflammatory root resorption (OIIRR) (1, 2). OIIRR is a pathological side effect after orthodontic treatment that is usually detected by a routine x-ray examination (3). OIIRR may occur in any or all teeth; however, it mostly occurs in the maxillary incisors. Because of the insidious nature of its onset, it is often found with a large lesion. Although the pathological process has been studied extensively, the true mechanism remains unclear. OIIRR is recognized as a destructive process of dentin by the activity of odontoclasts. It is currently believed that the risk factors of OIIRR include two parts: one is the origin of treatment such as lengthening of treatment and heavy orthodontic force. The other is the origin of patients such as genetics, abnormality of root form, and history of tooth trauma (4).

## Case report

### History

A referral was made to the Endodontic Department of Xi'an Jiaotong Hospital by the patient's orthodontist. A woman in her 20s had a chief complaint of recurrent swelling in the gum of the upper incisors.

**Figure 1:**
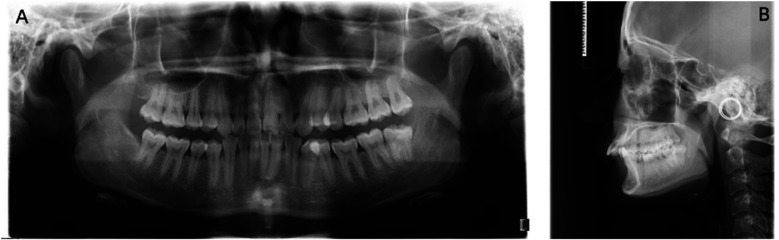
Radiological examination performed before orthodontic treatment. **(A)** Orthopantomogram. **(B)** Cephalometric radiograph.

**Figure 2:**
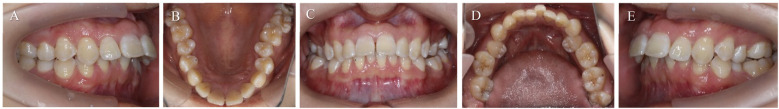
Intro-oral photographs taken before orthodontic treatment. **(A)** Right lateral view. **(B)** Maxillary occlusal view. **(C)** Frontal view. **(D)** Mandibular occlusal view. **(E)** Left lateral view.

The patient received orthodontic therapy because of skeletal class II and Angle's Class II malocclusion. The radiological examinations performed before the orthodontic treatment are shown in Figure 1. The intro-oral photographs before the orthodontic treatment are shown in Figure 2. After 1 year, the incisor teeth began to develop recurrent abscess in the gum. After incision and drainage of abscess performed in the periodontal department, there was no remission of symptom. The patient was referred for endodontic advice. The patient was in good health, with no significant past or present illnesses. She reported no history of any dental trauma, and she had a non-contributory personal and family history. The patient has good oral hygiene habits and is presently in good periodontal condition, with no signs of gingivitis or periodontitis, and demonstrated good compliance during the treatment.

### Assessment

**Figure 3:**
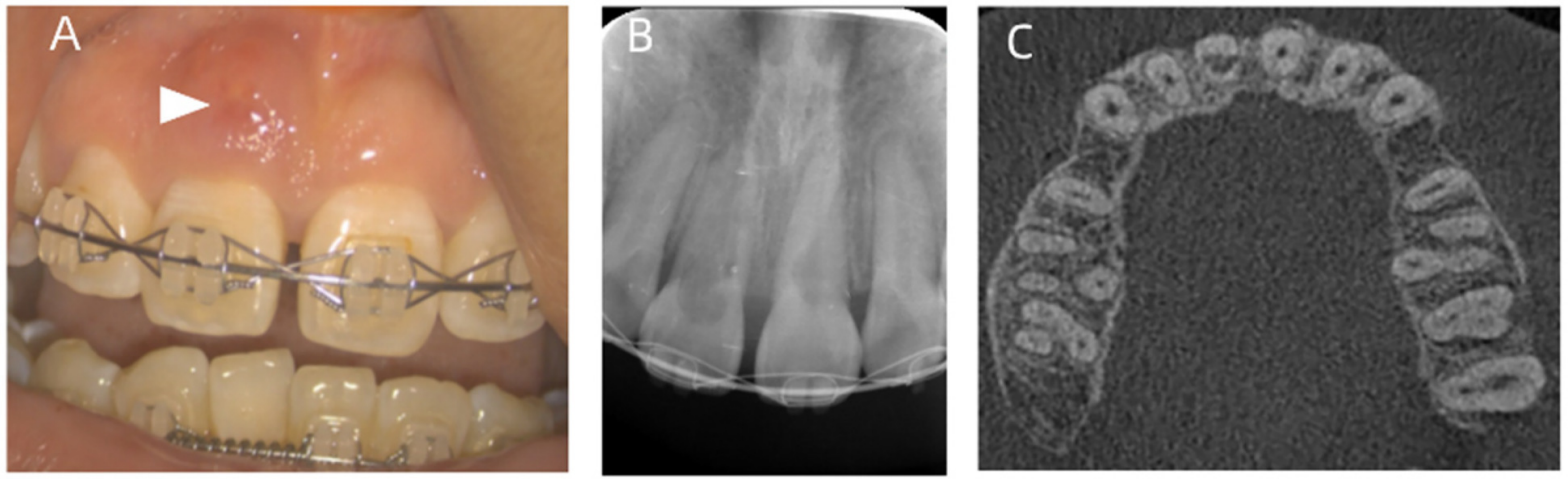
Oral examinations performed before root canal treatment. **(A)** Intro-oral photographs before root canal treatment. The white arrow points to the sinus on buccal mucosa. **(B)** An x-ray shows that in the region of the pulp chamber to the apical quarter of the root, an irregular low-density image area is observed in the upper right central incisor. In the root canal of the upper left central incisor, a semicircular low-density area is observed. **(C)** A CBCT examination of the initial dental status. The pulp chamber and the root canal of the upper right central incisor are absorbed, the walls of the root canal are thin, and the thickness is about 1 mm.

An extraoral examination revealed that the patient had a symmetrical face without swelling. An intraoral examination revealed that there was no color change and caries in the upper right central incisor. A swelling of the gingival mucosa with pus and blood spilled at 1/3 of the neck on the labial side of the upper right central incisor (Figure 3A). The depth of the periodontal probing was 4 mm. The cold test response was positive, and on percussion test, the patient reported mild pain. The mobility of the upper right central incisor was class I. The upper left central incisor displayed no caries, a cold test showed positive results, and the gum was normal. The mobility was class I.

A radiographic assessment by x-ray showed that in the region of the pulp chamber to the apical quarter of the root, an irregular low-density image area was observed in the upper right central incisor (Figure 3B). A CBCT showed that the pulp chamber and the root canal of the upper right central incisor were absorbed, the walls of the root canal were thin, and the thickness was approximately 1 mm (Figure 3C). An absorptive perforation was observed 3 mm below the cervical margin, and the labial alveolar bone was absorbed. A periapical low-density image was observed. An x-ray showed a semicircular low-density area in the root canal of the upper left central incisor.

### Treatment

Based on the above information, we diagnosed the patient with internal resorption and periapical abscess in the upper right central incisor and an internal resorption in the upper left central incisor. The clinical condition was explained to the patient. Once a diagnosis of internal resorption has been made, the clinician has several treatment options available, including tooth extraction. The patient will insist on saving the teeth; therefore, the treatment plan called for a suspension of orthodontic treatment and root canal treatment (RCT) of the infected upper central incisors.

**Figure 4:**
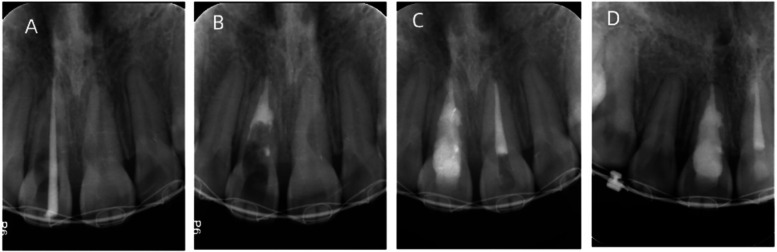
X-ray examination of the root canal treatment and a 3-year follow-up. **(A)** Working length determination. An x-ray shows that the working length is appropriate. **(B)** MTA is used for apical barrier and perforation repair. An x-ray shows that the apical 4 mm segment is tightly sealed with MTA as an apical barrier, and the lateral root perforation is repaired with MTA. **(C)** An x-ray taken right after root canal filling. The x-ray shows satisfactory root canal filling of the upper left central incisor and upper right central incisor. **(D)** At a 3-year follow-up, an x-ray reveals favorable prognosis in the upper left central incisor and upper right central incisor, with no periapical radiolucency observed.

Orthodontic treatment was immediately suspended. The incisors were isolated with a rubber dam, 4% of articaine (Bilan Company, France) under local anesthesia to open the pulp. Access to it was achieved on the lingual aspect of the crown. After reaching the pulp chamber, the roof was removed. The coronal aspect of the canal was opened by using a Gates Glidden. Then, we located canals with #08 and #10 K-files (Dentsply/Maillefer) and found that the apical stop was large, due to the root resorption. Therefore, the working length was difficult to be achieved by using a Root ZX apex locator (J. Morita, Tokyo, Japan). In addition, there was perforation in the defect of the root wall in the upper right central incisor. The working length was assessed radiographically (Figure 4A). Instrumentation was conducted by using WaveOne sequence files to 40/0.08 (Dentsply/Sirona, America). Sodium hypochlorite (2.5%) and 17% ethylene diamine tetraacetic acid (EDTA) were alternately used as an intracanal irrigating solution. After rinsing the root canal with ultrasound, a final rinse with a sterile normal saline solution was performed. The canal was dried with sterilized paper points (GAPADENT, Korea). The perforation on the root canal wall was repaired by using mineral trioxide aggregate (MTA). We performed the apical barrier technique using 4 mm lengths of MTA placed by the hand method under the microscope (5). The effect of the fillings was assessed on a radiograph (Figure 4B). Repair of the perforation can also be seen on the radiograph. After the repair, we used the continuous wave condensation technique (Beefill, VDW, German) to fill the remaining gaps in the root canal. Then, the coronal cavity was restored by using a resin composite. The upper left central incisor was located with #08 and #10 K-files (Dentsply/Maillefer) and the working length was established by using the Root ZX apex locator (J. Morita, Tokyo, Japan). The root canal was prepared by using WaveOne to 40/0.08, and 2.5% NaClO and 17% EDTA were alternately used to rinse the canal with sonic-powered EDDY tips (VDW, Germany); it was finally irrigated with a saline solution and dried. The iRoot SP (Innovative BioCeramix, Canada) was used as a root canal sealer combined with the warm vertical condensation technique. Lastly, resin was used to restore the coronal cavity. The final x-ray of filling is shown in Figure 4C.

**Figure 5:**
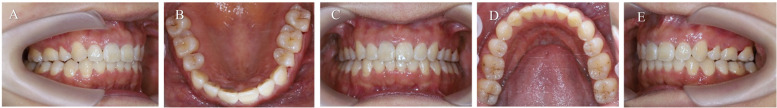
Intro-oral photographs taken after orthodontic treatment. **(A)** Right lateral view. **(B)** Maxillary occlusal view. **(C)** Frontal view. **(D)** Mandibular occlusal view. **(E)** Left lateral view.

After 2 weeks, the patient was reviewed. On reviewing the patient, it was found that the mobility of the teeth was normal and there was recovery of the gum’s condition. The periodontal probing depth of the upper right central incisor was 2–3 mm. At the 8-month follow-up, there were no clinical signs or symptoms. At the 3-year follow-up, the teeth were asymptomatic. The periapical radiograph revealed that the central incisors had no radiolucent lesions in the periapical tissues (Figure 4D). After orthodontic treatment was completed, the gingiva and periodontal tissues appeared normal on intraoral view (Figure 5). The patient reported no pain and expressed high satisfaction with the treatment. The teeth were well saved.

## Summary

Long treatment duration and the use of excessively heavy orthodontic forces are two risks for internal resorption. We inferred that orthodontic force caused the internal tooth resorption in this case based on three pieces of evidence. First, potential alternative causes (e.g., trauma, caries, periodontal infection, or idiopathic factors) were ruled out through clinical history, radiographic examination, and patient-reported data. Second, internal resorption was radiographically confirmed after the onset of orthodontic treatment, with no signs of pathology. Third, the patient complained of obvious continuous pain of the tooth over a period of 7 days after the return visit. The patient also complained of mobility during active tooth movement, which further corroborated the force intensity. Heavy force can impede the blood circulation of the pulp tissue, causing pulp congestion. This gradually develops into pulpitis and eventually pulp necrosis. Inflammatory factors are released in the pulp tissue, further leading to the formation of an abscess (6).

Even though severe orthodontically induced inflammatory root resorption can be a clinical challenge, timely treatment and the application of bioactive materials as a means of promoting mineralized tissue deposition and healing still provide clinicians with a treatment option for preserving affected teeth. It has been reported that with panoramic or periapical radiographs, OIIRR is usually less than 2.5 mm (7). However, in this patient case, the range of internal absorption was large, and even caused root perforation of the upper right central incisor. This is classified as severe resorption according to grade scales (8, 9). OIIRR is something unusual and can be difficult to identify at an early stage, so it is important to minimize the etiologic factors during orthodontic procedures. It is now clear that heavy force increases the incidence and severity of OIIRR, and that a 2 to 3-month treatment pause (with a passive archwire) decreases further resorption (8). In this case, the patient had already developed severe periapical symptoms, which were suspected as a result of orthodontic treatment, so the first step was to suspend orthodontic treatment. Considering that the patient had a strong desire to preserve the teeth, we devised a root canal treatment plan. Access cavity preparation should be conservative, preserving as much dentine as possible, and should avoid further weakening of the already compromised tooth (10). Since the teeth in this patient had actively resorbing lesions, and the patient was young, bleeding from the inflamed pulpal and granulation tissues might be substantial, which may affect visibility. The shape of the resorption defect usually renders it inaccessible to direct mechanical instrumentation. After opening the pulp, we used a small spoon excavator to remove the granulation tissue, and then rinsed the pulp cavity with a large amount of (3%) sodium hypochlorite in order to stop the blood supply of the clastic cells. The onset and development of root resorption are associated with risk factors related to orthodontic treatment, such as the treatment duration, the magnitude of the force application, the direction of the tooth movement, the mode of force applied (continuous or intermittent), and so on. Therefore, OIIRR is not completely preventable. In this case, the most informative study is CBCT, which reveals the character of the resorptive area, i.e., the exact size and dimension of the resorptive area, the extent of resorptive lesions, and the presence of root wall perforation, thus allowing dentists to draw an appropriate treatment plan (11). It is also reported that teeth with a history of trauma are more likely to suffer from root resorption during orthodontic treatment (12). Treatment for root resorption depends on the etiology. One of the key methods is to use sodium hypochlorite for the chemical dissolution of pulp tissue. The use of EDDY helps to activate sodium hypochlorite and facilitates the solution to penetrate all areas of the root canal. This ensures a chemomechanical debridement of the root canal system in depth (13). To seal the root resorption lacuna and the open apical foramen, bioactive cements MTA was applied in obturation. MTA is composed of silicate cement and bismuth oxide and is confirmed to have a novel biocompatibility, superior sealing properties, and antibacterial characteristics. It is also considered to be bioactive and well tolerated by periapical tissues (14). In addition, it has been shown to be effective in repairing root resorptions since it has bioactivity-inducing hard tissue formation, self-adhesion to dentine, and less shrinkage (15, 16). In a humid environment, MTA undergoes a curing reaction, reacting with water to form calcium silicate hydrate gel and generating calcium hydroxide at the same time. In this patient case, MTA was used for the apical barrier technique and perforation repair. The 3-year follow-up showed that the treatment was successful, with complete regression of the resorption and the absence of any signs or symptoms. Currently, biodentine is widely used as a new calcium silicate–based material for filling resorptive defects (17). However, due to the stipulation that overloading should be avoided before complete solidification and due to its high cost, MTA was chosen in this case.

## Conclusion

Even though severe orthodontically induced inflammatory root resorption can be a clinical challenge, timely treatment and the application of bioactive materials as a means of promoting mineralized tissue deposition and healing still provide clinicians with a treatment option for preserving affected teeth. We acknowledge that a single case limits drawing broader conclusions and therefore future studies with larger cohorts are needed.

## Data Availability

The original contributions presented in the study are included in the article/Supplementary Material, and further inquiries can be directed to the corresponding authors.
